# Identification of conserved splicing motifs in mutually exclusive exons of 15 insect species

**DOI:** 10.1186/1471-2164-13-S2-S1

**Published:** 2012-04-12

**Authors:** Patricia Buendia, John Tyree, Robert Loredo, Shu-Ning Hsu

**Affiliations:** 1 INFOTECH Soft, Inc, Miami, USA; 2 Masterschool of Informatics, University of Amsterdam, Amsterdam, Netherlands; 3 School of Computing & Information Science, Florida International University, Miami, USA; 4 United Biomedical, Inc., Asia, Hsin-Chu, Taiwan (R.O.C

## Abstract

**Background:**

During alternative splicing, the inclusion of an exon in the final mRNA molecule is determined by nuclear proteins that bind cis-regulatory sequences in a target pre-mRNA molecule. A recent study suggested that the regulatory codes of individual RNA-binding proteins may be nearly immutable between very diverse species such as mammals and insects. The model system *Drosophila melanogaster *therefore presents an excellent opportunity for the study of alternative splicing due to the availability of quality EST annotations in FlyBase.

**Methods:**

In this paper, we describe an *in silico *analysis pipeline to extract putative exonic splicing regulatory sequences from a multiple alignment of 15 species of insects. Our method, *ESTs-to-ESRs *(E2E), uses graph analysis of EST splicing graphs to identify mutually exclusive (ME) exons and combines phylogenetic measures, a sliding window approach along the multiple alignment and the Welch's t statistic to extract conserved ESR motifs.

**Results:**

The most frequent 100% conserved word of length 5 bp in different insect exons was "ATGGA". We identified 799 statistically significant "spike" hexamers, 218 motifs with either a left or right FDR corrected spike magnitude p-value < 0.05 and 83 with both left and right uncorrected p < 0.01. 11 genes were identified with highly significant motifs in one ME exon but not in the other, suggesting regulation of ME exon splicing through these highly conserved hexamers. The majority of these genes have been shown to have regulated spatiotemporal expression. 10 elements were found to match three mammalian splicing regulator databases. A putative ESR motif, GATGCAG, was identified in the ME-13b but not in the ME-13a of *Drosophila N-Cadherin*, a gene that has been shown to have a distinct spatiotemporal expression pattern of spliced isoforms in a recent study.

**Conclusions:**

Analysis of phylogenetic relationships and variability of sequence conservation as implemented in the E2E spikes method may lead to improved identification of ESRs. We found that approximately half of the putative ESRs in common between insects and mammals have a high statistical support (p < 0.01). Several *Drosophila *genes with spatiotemporal expression patterns were identified to contain putative ESRs located in one exon of the ME exon pairs but not in the other.

## Background

Alternative splicing is widespread during gene expression and has been studied thoroughly in mammals and insect species in recent years. The proportion of DNA that codes for proteins is greatly reduced in eukaryotes, but the number of ways in which the coding blocks called exons are combined to form new proteins is surprisingly large [1-3]. During transcription, intervening sequences (introns) in pre-mRNA molecules are spliced out by the spliceosome machinery and then exons are joined together to form mature mRNA molecules. Alternative splicing occurs when the exons of the RNA molecules produced by transcription of a gene are reconnected in multiple ways. Little is known about why one exon is chosen over another under specific circumstances. Pairs of exons that are never included together in the final mRNA transcript are called mutually exclusive (ME) exons. During the processing of pre-mRNA transcripts, accurate discrimination of exons and introns requires additional regulatory elements in addition to splice site (SS) signals at the 5'- and 3'-ends of exons. These conserved motifs have been termed exonic and intronic splicing regulatory sequences (ESRs/ISRs) because they occur in the exons or introns of a gene sequence and can be subdivided into exonic and intronic splicing enhancers (ESEs/ISEs) and exonic and intronic splicing silencers (ESSs/ISSs) that activate or repress splicing, respectively [4,5]. Since these regulatory sequences are relatively short, usually 4-18 nucleotides, most ESR studies have focused on hexamers [6-8].

A recent study suggested that the sequence specificity of RNA-binding proteins that target splicing regulatory sequences is conserved from insects to mammals [9]. Several groups have used microarrays in conjunction with manipulation of splicing regulator expression or crosslinking immunoprecipitation (CLIP) of splicing regulators to identify their indirect or direct targets [10,11]. Such studies provide the most valuable data for dissecting alternative splicing regulation centered on one splicing regulator of interest. Several systematic computational approaches combined with *in vivo *or *in vitro *selection methods have been employed to identify motifs in genomic sequences [7,8,12-15]. Bioinformatics approaches such as comparative genomics analysis have provided significant sequence and functional insights into the regulatory sequences that occur within exonic regions of a transcript. In combination with motif analysis, one can further study motif enrichment in a group of tissue-specific alternative exons [16,17]. ME exons have been shown to be expressed in different tissues and cell lines or at different stages during development [2,18,19]. A recent study showed that most identified exonic regulatory elements were found to contribute to the alternative splicing between two tissues, while some were important in multiple tissues [20]. A systematic analysis of complete alternative splicing events in a recent study focused on the identification of exon skipping and ME splicing events [21].

Recently, several alternative splicing databases have been built from abundant expressed sequence tags (ESTs) [22-25]. One of these databases, *the Drosophila melanogaster *Exon Database (DEDB), uses de-Bruijn graphs constructed by matching together similar EST segments [24,26]. DEDB contains entire gene sequences in splicing graph format, which are used to represent different splicing events [24]. To generate the splicing graphs, EST transcripts were clustered on the basis of overlapping genomic positions they occupy. Exons and introns with identical start and end positions were merged into nodes and connections respectively. The nodes were then linked via connections to form the complete splicing graph for a gene and stored as xml files [24].

## Methods

We propose a new method, ESTs-To-ESRs (E2E), that uses splicing graphs from the *Drosophila melanogaster *Exon Database (DEDB) to identify mutually exclusive (ME) exons and subsequently finds putative ESR motifs by looking for conserved fragments in a multiple alignment of these ME exons. Our focus was on ME exons as we were interested in identifying tissue-specific *cis*-regulatory splicing sequences, which are thought to regulate the inclusion of one exon over another depending on the tissue in which they reside. We applied E2E to 15 species of insects, including 12 Drosophila species, *Apis mellifera, Tribolium castaneum *and *Anopheles gambiae*. We compared 100% conserved motifs with those obtained using the E2E sliding window spikes method and we compared the insect exonic motifs with those published for mouse and human [6-8]. Finally, we also looked at some mutually exclusive exons in the *Drosophila melanogaster *and compared our findings with those from a recent study [27].

The E2E method uses an *in silico *analysis pipeline that includes three steps: (1) identification of mutually exclusive exons in the *Drosophila *splicing graphs, (2) acquisition and pre-processing of the multiple alignment of mutually exclusive exons and of the phylogenetic tree of 15 species of insects, and (3) a sliding window comparative analysis that identifies putative ESRs.

### Identification of ME exons

A recent study [9] has demonstrated the high degree of conservation for splicing regulatory sequences in different species. This high degree of conservation further supports similar regulatory mechanisms of RNA splicing and the biological importance of protein products containing mutually exclusive exons. We focused on mutually exclusive exons because these have been shown to have evolved from exon duplications in 60% of the cases and are believed to be a major route for generating functional diversity during the evolution of multicellular eukaryotes [28-30]. Exon duplications and therefore ME exons represent the origin of alternative splicing and deserve special attention. In order to extract evolutionarily conserved ME exon pairs in insect species, we started with the identification of ME exon pairs from the transcriptome of *Drosophila melanogaster*. The *Drosophila melanogaster *Exon Database (DEDB) contains splicing graphs for gene sequences constructed from EST transcripts [24]. Splicing graphs were downloaded as xml files and used to extract additional information, such as the type of splicing events: intron retention, cassette exon splicing, alternative termination, among others. We focused on the identification of cassette exons. A cassette exon event involves an exon that is sometimes included in a transcript and sometimes absent.

**Figure 1:**
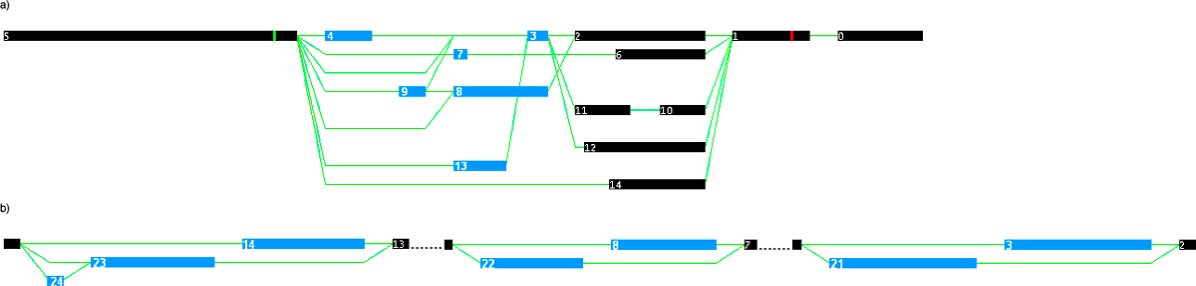
**Splicing graphs for *Drosophila melanogaster *genes**. a) Splicing graph for the *Spaetzle *(*spz*) gene (CG6134). b) Simple splicing graph for the *N-Cadherin *(*CadN*) gene (CG7100).

In particular, splicing graphs in xml format were used to identify mutually exclusive exons. The DEDB online database visually displays a splicing graph as exon and intron connections for alternative gene transcripts (See Figure 1). Each splicing graph represents all observed contiguous mRNA transcripts for one single gene.

As a first step in the identification of ME exons, splicing graphs containing the phrase "<cassette_exon>" were downloaded from DEDB in xml format. The xml files were parsed and further classified by the flybase gene symbol, node id, start and end position of all exons and introns, and the entire sequences of all exons (nodes) and introns (connections) were collected.

The sample splicing graph in Figure 1a shows several types of alternative splicing events. In the graph, individual transcripts are represented as a left to right traversal through a set of connected nodes. Cassette exons involved in mutually exclusive splicing are represented as blue squares (exons 3, 4, 7, 8, 9, 13). A series of cassette exons (e.g. 6, 10, 11, 14) that overlap with exons 2 and 12 which are connected to either exon 8 or 3 are excluded as ME exon candidates (see 'Algorithm for Identifying ME Exons' below). In order to be considered mutually exclusive, the cassette exon must appear in one transcript and be absent in another, and a different exon must be chosen instead. Exons 4 and 9, for example, are mutually exclusive exons. Their sequences appear at different non-overlapping positions in the genome and they appear in different transcripts, never together. The horizontal lines represent connections to another exon corresponding to the DNA sequence that separates them in the gene sequence, including introns or other exons not present in a transcript. Identifying ME exons requires more than just searching for exons that are not connected. Exons 8 and 3 in Figure 1a are overlapping exons and therefore not mutually exclusive. They share the same sequence for some of their length, but exon 3 starts later than exon 8. Exons 4 and 8 are not mutually exclusive even though they are not connected (they are not on the same path) because exons 4 and 3 appear on the same transcript and exon 3 overlaps with exon 8.

### Algorithm

The algorithm for identification of ME exon pairs uses as input a splicing graph for a gene containing cassette exons. The terms node (in the graph) and exon (in the sequence) will be used interchangeably in the following paragraphs. The graph is stored as a directed acyclic graph (DAG) to allow for efficient Depth first searches (DFS). Figure 1 does not show the direction of the edges but there is an implied left to right traversal order for all paths through the graph.

The first step in the algorithm is to construct a list of all cassette exon pairs by identifying the DEDB exons with the 'cassette exon' annotation and initialize them as being candidates for ME exon pairs. Then one by one the pairs of ME exon candidates are excluded from the list if they do not satisfy a set of rules. A DFS traversal is used to exclude connected exons. This approach discards exon pairs that could potentially occur in the same transcript, based on the observed EST evidence. For example, exons 22 and 21 in Figure 1b are not considered mutually exclusive because they reside in separate splicing subgraphs connected by regular exons (black squares). In the algorithm described below, each pair will be flagged with a status: "m" for mutually exclusive, "o" for overlap, "c" for connected, "t" for rule three, "f" for rule four.

#### Algorithm for identifying ME exons

Input: Splicing graph *S *with *n *cassette exon nodes (*S_i _*for *i *= *1,..,n*) from a single gene

Output: List of mutually exclusive exon node pairs

1. Initialize a list of cassette pairs with

• (rule 1) "o" if they overlap or "m" as ME candidates (e.g. Exons 3 and 8 overlap)

2. Loop through the cassette exon list

• (rule 2) Run a Depth-first search (DFS) for each cassette exon a and for each cassette exon b encountered, flag the pair (a, b) with "c" (e.g. Exons 9 and 8 are connected)

3. For all "m" exon pairs

• (rule 3) if exon a in a pair (a, b) overlaps with an exon c in another pair (c, b) which has been marked as "c", mark (a, b) as "t" (e.g. Pair (9,13) are not ME exons because 13 overlaps with 8 and 8 is connected to 9)

• (rule 4) if an exon a in a pair (a, b) overlaps with an exon c in another pair (c, b) marked as "m", then if length(a)<length(c) mark pair (a, b) as "f", else mark (a, c) as "f" (e.g. Pairs (4,7) and (4,13) are ME exon pairs, but for our analysis purposes because 7 and 13 overlap we only pick (4,13))

### Retrieval of multiple alignment of ME exons and a phylogenetic tree of 15 insect species

To obtain the multiple sequence alignment of ME exons, the *Drosophila melanogaster *genome sequence was downloaded from the UCSC ftp server: http://hgdownload.cse.ucsc.edu/downloads.html. The cassette exons of 76 genes were mapped onto the downloaded *Drosophila melanogaster *genome using BLAST. Based on the scaffolds created by the downloaded *Drosophila melanogaster *genome, reading frames were created using the node id, chromosome, start- and end-positions of the cassette nodes in the splicing graphs. These reading frames created starting points, as well as information regarding the length of the nodes. ESTs that were used to generate the splicing graphs were used as guidelines to verify correctness of exon start, end positions and orientation. Manual inspections of the data lead to the removal of incongruent cassette exons. Multiple alignments of the 269 cassette exon containing 15 different insects, incuding 12 Drosophila species*, Apis mellifera*, *Tribolium castaneum *and *Anopheles gambiae*, were downloaded from the UCSC ftp server in MAF format. The MAF alignments were converted into Fasta alignments using a Perl script which generates the reverse complement for the exons transcribed from the other strand and also verifies that the downloaded sequences cover the exons in question. The multiple alignments from UCSC were chosen over other alignment options (e.g. BLAST) to ensure that the sequences used to extract ESRs were the homologous sequences of the genes from individual species as there are several repetitive extracellular domains for some genes (e.g. homologous gene N-Cad2 and N-Cad family genes). A phylogenetic tree of the 15 insect species was also obtained from the UCSC Drosophila database. The UCSC tree was generated with phyloFit and phastCons to estimate conserved and non-conserved branch lengths [20]. The UCSC tree was the preferred choice as it represents the consensus based on the whole genome sequences of all 15 species and a self-generated tree would have been based on a limited number of sequences.

### Identification of ESRs

The aligned ME exon sequences of 15 insects were searched for conserved words of lengths 5-9 bp as ESRs are relatively short and recent studies focused mainly on hexamers [7,8]. Two different approaches were used to identify these conserved words as putative ESRs:

1. Words that were within 100% conserved regions regardless of flanking sequences

2. Words that were significantly more conserved than flanking sequences in phylogenetic clusters using the Welch's t test

Of special interest were motifs that appeared in one ME exon but not the other within a ME exon pair.

### 100% conserved regions

We used a simple sliding window algorithm to scan all columns of an exon multiple alignment with an initial window length of 5 bp. If no gaps were present in that window and the conservation was 100%, the size of the window was increased by one until a non-conserved column was found. The *Drosophila melanogaster *nucleotide sequences corresponding to the conserved segments in ME exons were saved to a file. In a second step, all words of length n, with n = 5 to 9, were extracted from the conserved segments. The frequency of the words and their location within an exon were subsequently determined.

### Conservation spikes in phylogenetic clusters

**Figure 2:**
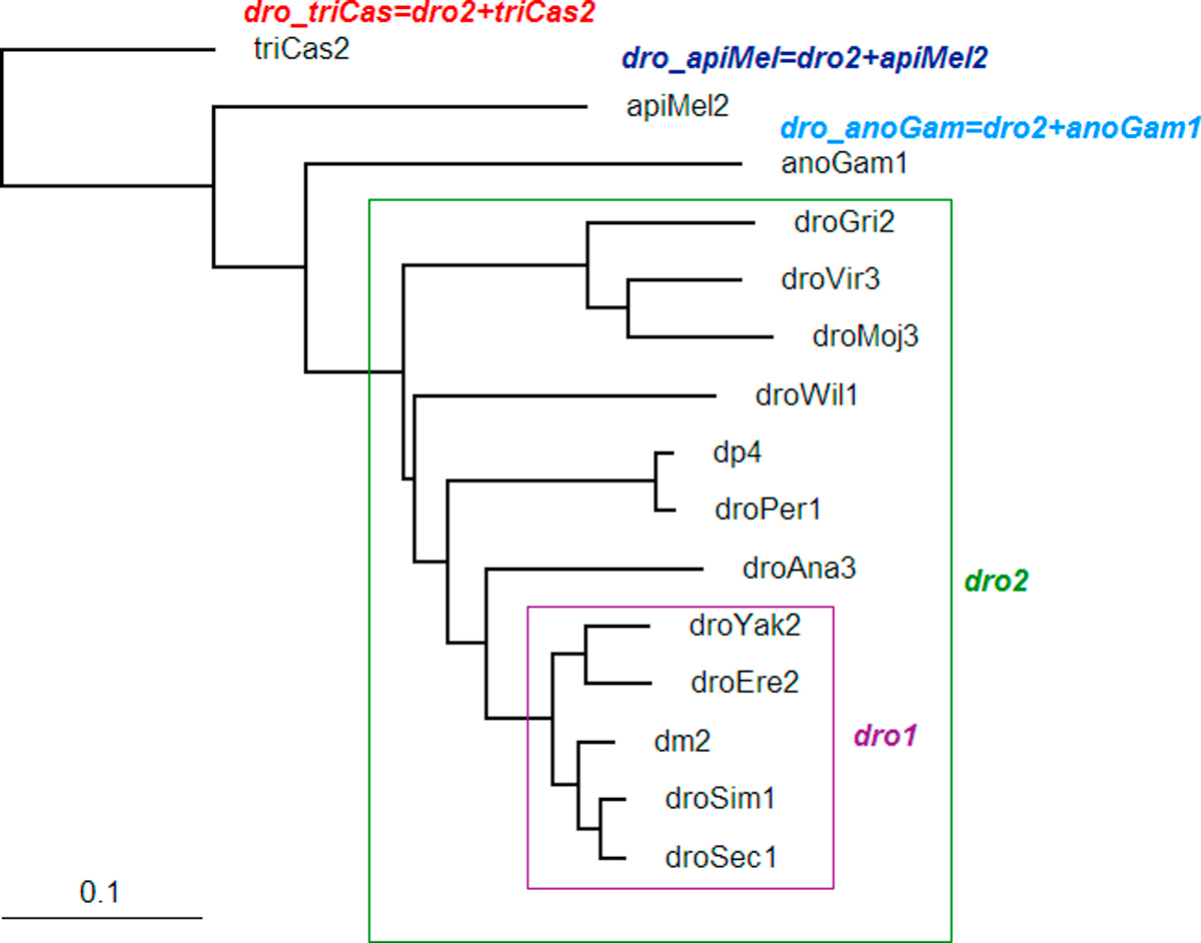
**Phylogenetic tree showing 5 different clusters of related insect species**.

This method implemented a sliding window approach along the multiple alignments of exons. A conserved segment is not required to be 100% conserved but has to be significantly more conserved than the neighboring segments and above a predefined phylogenetic conservation threshold *q^+^*. An appropriate value for *q^+ ^*can be found in the evolutionary conservation scores computed from a phylogenetic tree of the 15 insect species. Additionally, phylogenetically related insect species were grouped into overlapping clusters. The tree topology was used to separate insect species into 5 phylogenetic clusters as shown in Figure 2. *q^+ ^*is therefore calculated once for each cluster in the phylogenetic tree and compared to the conservation score of hexamers in the sequence alignments.

Phylogenetic tree distances were used to calculate the tree conservation scores. The "branch lengths" (expected number of substitutions per site) were replaced with tree conservation scores (proximities) in the phylogenetic tree. The tree conservation score *q *(or the proximity of a species to its ancestor) was defined as the probability that any given base which is not under selection has not mutated in the time separating the ancestor and the descendant. If the tree specifies the number of synonymous substitutions per site s, then as suggested in [31], the proximity may be reasonably approximated as

$$q=e^{-s}$$

Let *q^+ ^*= *q *+ *a*, be the global conservation score threshold for each cluster, with *a *= *1*-*q*(dro1). For each window, we compared the conservation score, which is in the interval 0[1], with '1' indicating 100% conservation, to the global conservation score *q^+^*. The dro1 cluster was given a threshold of 1 because of the close evolutionary relatedness of the species in this cluster. In our calculated conservation scores (Table 1), *a *= 0.038892.

**Table 1:** Tree conservation scores

Cluster	Tree distance	*q*	*q^+^*
**dro1**	0.039669	0.961108	1
**dro2**	0.21	0.810584	0.849477
**dro_anoGam**	0.191225384	0.825946	0.864839
**dro_apiMel**	0.231922	0.793008	0.8319
**dro_triCas**	0.324132615	0.723154	0.762047
**all**	0.3252756	0.722328	0.761221

A putative ESR was identified if a particular window showed a spike in the conservation score. That is, when the window had a conservation score no less than the *q^+ ^*threshold and was flanked by columns with a lower average conservation score. The conservation score at position × in the multiple alignment for a window of size w is given by:

$$c\left[x\right]=\frac{1}{w}\overset{w}{\underset{i=1}{\sum }}\frac{m_{i}}{S}$$

where *m_i _*is the number of matches in column *i *and *S *the number of species, 15 in our case.

**Figure 3:**
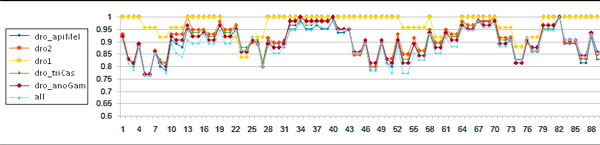
**Graph showing spikes in conservation scores for 5 phylogenetic clusters and the complete multiple alignment (all)**.

A spike in conservation scores for several clusters and most prominently the dro_anoGam cluster can be observed in Figure 3, between positions 13-22, between 31-40, 64-70, and 79-82. The statistical significance of such spikes was assessed with the Welch's t test for each cluster. Below, we present the spike identification rules:

1. For a window's conservation score at position *x*, c[*x*], the following should be true:

• c[*x*] ≥ *q^+ ^*for a particular cluster

• c[*x-w*] < c[*x*] > c[*x+w*] with w as the window length

2. A spike is deemed significant if *p_x-w _*< 0.05 and *p_x+w _*< 0.05, with *p_x-w_*, the left p-value for a t-test comparing c[*x-w*] with c[*x*] and *p_x+w_*, the right p-value for a t-test comparing c[*x+w*] with c[*x*]

In order to conduct the t-test, the proportions were transformed into arcsine values to comply with the assumption of normality for t-tests as proportions have fixed limits 0-1. The arcsine values and standard deviations were used to calculate the Welch's t statistic. Spikes for windows of size 6 were identified by using a sliding window approach with a step size of 1.

For spikes at the start and end of sequences (for which only a one sided p-value can be calculated) we increased the q^+ ^threshold by 0.1. The p-value for the difference in spike magnitude between windows of length w starting at positions x1 and x2 with standard deviations s1 and s2 was computed using the Welch's t-test:

$$t=\frac{c\left[x_{1}\right]-c\left[x_{2}\right]}{\sqrt{\frac{s_{1}^{2}}{w}+\frac{s_{2}^{2}}{w}}}$$

### Multiple testing correction

The false discovery rate (FDR) multiple comparison correction developed by Benjamini and Hochberg [32] was applied to the data to control the false positive rate. Unlike earlier analysis, which only considered windows with p-values < 0.05, all p-values for every comparison at every positions of the alignment across all 269 cassette exons were considered for the multiple comparison correction. Adjusted p-values and false discovery rates were computed separately for each left and right t-test window comparison regardless of the phylogenetic cluster.

## Results

### 100% conserved words

When the 100% conservation rule was applied without consideration of surrounding bases, 482 words of length 5- 22 bp were found in the multiple alignments of 269 cassette exons from 76 genes of 15 insect species. Words of 6 bp in length or more were not observed more than 1 to 3 times. The most frequent word of length 5 bp appeared 13 times in different exons: ATGGA. ATG[A, C, G]A was a frequent motif occurring 9, 9, 13 times, respectively. The reverse (but not the reverse complement) of the ATGGA sequence often appears in the 5' splice site. However, this particular conserved sequence is not found near the splice site in any of those 13 occurrences. None of the most frequent 100% conserved words matched the words found using the conservation spikes method described below.

### Spikes in sequence conservation

A list of 799 statistically significant "spike" hexamers was found by the E2E method. About half or 379 elements had a left or right p-value < 0.01. Only 83 elements had both left and right side spike magnitude p-values < 0.01. We were interested in identifying elements in the last list that appear in one mutually exclusive exon but not in the other, investigating how frequently this occurs and if possible, deducing their impact on tissue-specific splicing. Of these highly significant elements only five genes had ME exons where the elements in one ME exon differed from those in the other: SNF4Aγ (CG17299, FBgn0025803), Doa (CG42320, FBgn0259220), Zasp52 (CG30084, FBgn0083919), Imp (CG1691, FBgn0030235), Pdp1 (CG17888, FBgn0016694) and l(3)82Fd (CG32464, FBgn0013576).

By applying the FDR multiple comparison correction, we found 189 hexamer, 22 heptamer and 7 octamer motifs with significant left or right corrected p-value < 0.05 (See "Additional file 1" for FDR corrected list of motifs and matches to other human and mouse regulatory element databases). A single motif had both a left and right corrected p-value < 0.05 as the FDR significance threshold amounted to an uncorrected p-value threshold of <0.0003. The motif was found in gene Zasp52 (CG30084, FBgn0083919). Among the list of FDR significant hexamers, 11 genes had significant spikes in one ME exon that did not appear in the other. Five of them appeared in the previous list: Pdp1, SNF4Aγ, Doa, Zasp52, l(3)82Fd, and six new genes were added to the list: heph (CG31000, FBgn0011224), par-1 (CG8201, FBgn0260934), bbc (CG6016, FBgn0033844), sdt (CG32717, FBgn0261873), cg1637 (CG1637, FBgn0030245) and CG6043(CG6043, FBgn0032497). Even though alternatively spliced transcripts have been annotated for all these genes in the Flybase, no information on the tissue-specific expression patterns of alternatively spliced transcripts is available.

In order to assess the relevance of the predicted cis-acting elements, we compared the sequence similarities between the predicted elements with predicted binding sites from splicing regulator prediction databases of mouse and human [6-8]. Among the 799 statistically significant "spike" hexamers, 155 of the predicted elements could be validated with the Wang 2009 database (84 with a left or right p-value < 0.01), 102 elements matched with predicted exonic splicing regulators from the Ast Lab, (52 with a left or right p-value < 0.01) [7]. Only 10 elements matched with all three databases, including elements predicted by the Burge Lab, which were available only within the Wang data set. A majority of these 10 elements have left (L) or right (R) side spike magnitude p-values < 0.01 (Table 2). Four of those appear close to a splice site and four are in the dro_anoGam cluster. 39 of the 189 hexamers in the FDR significant spikes list were also found in the Wang list. Sequence logos for the consensus sequences of the significant hexamers were drawn using WebLogo. No discernable frequency pattern was found in the complete list of 189 FDR significant hexamers nor among the 22 hexamers in common between the Ast lab and the FDR spikes list. Surprisingly, hexamers that were also found in the Wang list have a sequence logo (or frequency plot) that shows a marked preference for the A base at the first two positions.

**Table 2:** Drosophila exonic elements supported by mouse and human databases

Gene	Exon start	Length	Start	End	dm2*	L* p-value	R* p-value	Conservation	Cluster
CG8857	278	183	71	76	AAGAAG	0.0415	0.0072	1	dro_anoGam
CG18350	5931	950	777	782	TTTGTT	0.031	0.0067	0.821	all
CG17927	9426	121	50	55	AAGAAA	0.0013	0.0038	0.988	all
CG3937	12352	510	470	475	GAAGAA	0.0332	0.0096	0.987	dro_triCas
CG12090	7282	114	109	114	AACCAG	0.0078	-	0.952	all
CG17762	12253	1355	1346	1351	AAGAAG	0.0003	-	1	dro_anoGam
CG32158	27126	906	901	906	CTGAAG	0.0009	-	0.952	all
CG7535	26856	71	27	32	AACATG	0.0105	0.0415	1	dro_anoGam
CG1725	20594	269	152	157	AAAGAA	0.0292	0.03	1	dro2
CG17762	12253	1355	1348	1353	GAAGAT	0.0233	-	1	dro_anoGam

*dm2 stands for Drosophila melanogaster, L and R stand for left/right spike increase/decrease respectively.

### ESRs in the *Drosophila N-Cadherin *gene

A recent study looked into the spatiotemporal differential expression of *the N-Cadherin *ME exons 7a and 7b, 13a and 13b, 18a and 18b of *Drosophila *and *Tribolium *(See Figure 1b) [27]. This study found that transcripts containing ME-13a are expressed only in the CNS, while those containing ME-13b are only expressed in the early mesoderm. Furthermore, the non-neuronal expression of ME-13b drops sharply before synapses begin to form in the embryos. Based on the significant spikes approach, we found a putative ESR motif, GATGCAG, in 13b for the complete alignment, close to the 3' splice site. A second ESR, AAATTG, was found in the dro_apiMel cluster, close to the 5' splice site. No significantly conserved spikes were found in exon 13a.

Exons 7a and 7b have less divergent protein sequences and our analysis found the conserved hexamer TGGGAT in the 7a exon and AAAGCCAG as a significant conservation spike near the 3' splice site for the dro_apiMel cluster in 7b.

Paralogous alternative exons 18a and 18b exhibit great sequence diversity from each other. No conservation spikes were detected in 18b. A conserved word of length 8, TGGGGCGA, appears in exon 18a in the dro_anoGam cluster but the L and R p-values are just below 0.05.

## Conclusions

We presented a bioinformatics workflow protocol to extract exonic splicing regulatory sequences (ESRs) from mutually exclusive exons of *Drosophila melanogaster*. We identified mutually exclusive exons in EST splicing graphs and used a phylogenetic conservation threshold to identify spikes in conservation through sliding window analysis of exon multiple alignments of 15 insect species, as a higher degree of nucleotide sequence conservation is frequently observed in the alternatively spliced exons and/or flanking introns than in the constitutive exons [7,33]. We looked at sequences with 100% nucleotide conservation, but found no hexamers among the cassette exons that appeared at high frequency and many conserved sequences were found to be part of highly conserved regions within the exons. Incorporating measures of phylogenetic relationships and variability of sequence conservation is a more qualitative approach for the identification of ESRs as it is possible to calculate the statistical significance of the identified motifs.

The E2E spikes method found 799 putative ESRs of which 379 elements had a left or right p-value < 0.01 and 83 elements had both left and right side spike magnitude p-values < 0.01. The FDR correction process resulted in 218 motifs with either a left side or right side corrected p-value < 0.05, but only 1 motif with both left and right corrected p-values < 0.05. The reason for this is that t-tests for a sample size of 6 will produce higher p-values leading to fewer significant motifs when a correction is applied. Among the 83 elements in the uncorrected list, we found five genes and among the 218 motifs in the corrected list we found 11 genes whose ME exons had putative ESRs that differed from ESRs in the other paired ME exons. It is believed that recognition of ESRs in the ME exons drives expression of mutually exclusive exons in a tissue-specific manner [17,18]. Unfortunately there is little information in the literature about the tissue-specific expression patterns of alternatively spliced isoforms of Drosophila genes, but we found information that linked certain genes to differing spatiotemporal expression. The gene Zasp functions in the formation of integrin adhesion sites and is active during different stages of development. At late embryonic stages, Zasp expression is particularly strong in mesodermal tissues such as visceral, pharyngeal, and somatic muscles [34]. Doa is expressed in ectoderm and mesoderm during early embryonic stages and in nervous system during late embryonic stages. SR and SR-like proteins can be phosphorylated by Doa [35,36]. Doa's ability to regulate activities of other splicing factors along with its many spliced isoforms may add more levels of complexity in gene expression regulation. Alternatively spliced sdt transcripts were shown to determine a developmental switch in mRNA localization in which apical transcripts were only found during early stages of epithelial development [37].

In the *N-Cadherin *gene, we observed that the dro_anoGam cluster which includes dro1 (or dro2) has a spike in 18a but not dro1 (dro2) itself, which may be due to divergent evolution of the flanking regions around the conserved word in more distant species. *In vivo *results from a recent study of the N-Cadherin gene, showed no differential subcellular localization between transmembrane domain isoforms containing exons 18a or 18b in Drosophila and showed the conserved *in situ *spatiotemporal expression patterns in Drosophila and Tribolium [27]. Although extensive knowledge of annotated spliced isoforms is available, more information on the *in situ *isoform-specific expression patterns would be required to validate this approach at a functional level.

A recent study identified and classified hundreds of alternative splicing events that are affected by one splicing regulator and showed that binding sites for the factor are conserved from insects to mammals [9]. Another study looked at alternative splicing patterns regulated by four Drosophila homologues of the mammalian hnRNP A/B family [38]. Both demonstrated conservation of splicing regulatory mechanism between insects and mammals. Thus, we compared ESRs predicted by the E2E method with splicing regulatory sequences from three other databases. 155 of the 799 predicted elements can be validated with the Wang 2009 database of human splicing regulatory sequences and 102 elements with the Ast Lab's list of human and mouse ESRs [7].

The regulation of RNA splicing is a complicated process as it involves the interplay of many splicing factors and their target sequences. Some exons are constitutively expressed while others are expressed in a tissue- and stage- specific manner. High-throughput sequencing of transcriptomes has recently demonstrated a high degree of alternative splicing, in up to 92~94% of protein coding genes in the human genome [39]. With a genome-wide approach, E2E method identified several ESRs within ME exon pairs based on the nucleotide sequence conservation among insect species. Some of these ESRs have been previously identified in mammalian splicing databases. Although the tissue-specific expression data of Drosophila genes are limited, a few genes containing these identified ESRs exhibit differential spatiotemporal expression patterns. This demonstrates that the E2E method is a powerful tool to help identify conserved splicing signals which might be of high importance in biological functions and shed light on the tissue- and stage-specific splicing regulations. Future work will include more in depth mining of the literature on tissue-specific splicing to validate ESRs found with the E2E method. We will also expand the E2E method to other alternatively spliced exons and introns and apply it to multiple alignments of alternatively spliced genes from human tumor tissue of different patients as it is known that aberrations in alternative splicing occur in many cancers [40].

## Competing interests

The authors declare that they have no competing interests.

## Authors' contributions

SNH collected and formatted the data and provided the biological background knowledge. SNH and PB designed the study. PB carried out the statistical analysis and drafted the manuscript. RL developed the splicing graph parsing algorithm. JT developed the ESRs comparative analysis method. All authors helped edit the manuscript and approved the final version.

## Supplementary Material

Additional file 1 **(PDF format) - FDR corrected list of motifs and matches to other human and mouse regulatory element databases**. The table shows the list of motifs with an FDR corrected p-value > 0.05. Motifs near the splice site were excluded. A star in L_sig or R_sig indicates a left or right side significant element. DM stands for Drosophila melanogaster, a 0 or 1 in the Wang Lab and Goren Ast Lab columns indicates a match with an element in those data sets. Click here for file
