# Supplementary material for: Identification of conserved splicing motifs in mutually exclusive exons of 15 insect species
Source: BMC Genomics. 2012 Apr 12;13(Suppl 2):S1. doi: 10.1186/1471-2164-13-S2-S1 (PMC3303723; doi:10.1186/1471-2164-13-S2-S1)
Supplement: Additional file 1 — (PDF format) - FDR corrected list of motifs and matches to other human and mouse regulatory element databases. The table shows the list of motifs with an FDR corrected p-value > 0.05. Motifs near the splice site were excluded. A star in L_sig or R_sig indicates a left or right side significant element. DM stands for Drosophila melanogaster, a 0 or 1 in the Wang Lab and Goren Ast Lab columns indicates a match with an element in those data sets. [file 1471-2164-13-S2-S1-S1.doc]

### Additional file 1 –FDR corrected list of motifs and matches to other human and mouse regulatory element databases

The table shows the list of motifs with an FDR corrected p-value > 0.05. Motifs near the splice site were excluded. A star in L_sig or R_sig indicates a left or right side significant element. DM stands for Drosophila melanogaster, a 0 or 1 in the Wang Lab and Goren Ast Lab columns indicates a match with an element in those data sets.

| Gene | Exon Start | Cluster | DM | Conservation | L_sig | R_sig | Goren Ast Lab | Wang Lab |
| --- | --- | --- | --- | --- | --- | --- | --- | --- |
| CG10279 | 2465 | dro2 | ATTGCGGT | 1 |  | * | 0 | 0 |
| CG10279 | 2465 | dro2 | GCGGTT | 1 |  | * | 0 | 0 |
| CG10693 | 24958 | all | TTCTTC | 0.989 |  | * | 1 | 0 |
| CG10706 | 48004 | dro_anoGam | ATGTGG | 0.987 |  | * | 0 | 0 |
| CG11081 | 3414 | all | AATTAT | 0.852 |  | * | 0 | 1 |
| CG11081 | 3414 | dro_triCas | ACAAGT | 0.917 |  | * | 0 | 0 |
| CG11081 | 3414 | all | TACAAG | 0.833 |  | * | 1 | 0 |
| CG11711 | 19399 | dro2 | AAGGAG | 0.944 |  | * | 0 | 0 |
| CG1637 | 2220 | dro_triCas | ACTGGT | 0.962 | * |  | 0 | 0 |
| CG1637 | 2220 | dro_triCas | CTGGTC | 1 |  | * | 0 | 0 |
| CG1637 | 2220 | dro_triCas | GGCTGG | 1 | * |  | 1 | 1 |
| CG1637 | 4328 | dro_triCas | AAAACG | 0.859 | * |  | 0 | 0 |
| CG1637 | 4328 | dro_apiMel | GACATG | 0.962 |  | * | 1 | 0 |
| CG17018 | 13831 | dro_triCas | AATTTT | 1 | * |  | 0 | 1 |
| CG1725 | 20594 | dro2 | GAAGCG | 1 |  | * | 0 | 0 |
| CG17299 | 33098 | dro_anoGam | AATGTC | 1 | * |  | 0 | 0 |
| CG17299 | 33098 | dro_anoGam | ATGTCG | 1 | * |  | 0 | 0 |
| CG17299 | 33098 | dro_anoGam | CAAAGA | 0.959 |  | * | 1 | 0 |
| CG17299 | 33098 | dro_anoGam | CAATGT | 0.986 | * |  | 0 | 0 |
| CG17299 | 33098 | dro_anoGam | CCAAAG | 0.946 |  | * | 0 | 0 |
| CG17299 | 33098 | dro_anoGam | TGTGAT | 0.987 |  | * | 0 | 0 |
| CG17299 | 60019 | dro2 | AATGCA | 0.986 | * |  | 1 | 0 |
| CG17299 | 60019 | dro_anoGam | TCGCTG | 0.958 | * |  | 0 | 0 |
| CG17299 | 60944 | all | ACAGCGG | 0.962 |  | * | 0 | 0 |
| CG17299 | 60944 | dro_triCas | AGACAG | 0.944 |  | * | 0 | 1 |
| CG17299 | 60944 | all | AGCAGC | 0.936 |  | * | 0 | 1 |
| CG17299 | 60944 | dro_triCas | CATCAC | 0.859 | * |  | 0 | 0 |
| CG17299 | 60944 | all | CCATCA | 0.821 | * |  | 0 | 0 |
| CG17299 | 60944 | all | GACAGC | 0.962 |  | * | 1 | 1 |
| CG17299 | 60944 | dro_triCas | GACAGCGG | 0.986 |  | * | 0 | 0 |
| CG17299 | 64525 | dro_anoGam | CAACAA | 0.986 |  | * | 0 | 0 |
| CG1765 | 45237 | dro_anoGam | AAAAATAC | 0.986 |  | * | 0 | 0 |
| CG1765 | 45237 | dro_anoGam | AAAATG | 0.972 | * |  | 0 | 0 |
| CG1765 | 45237 | dro_anoGam | AAATGTA | 0.958 | * |  | 0 | 0 |
| CG1765 | 45237 | dro_anoGam | AACGGC | 0.91 |  | * | 0 | 0 |
| CG1765 | 45237 | dro_anoGam | AATACTA | 0.972 |  | * | 0 | 0 |
| CG17762 | 12253 | dro2 | GCAAGA | 1 | * |  | 1 | 0 |
| CG17762 | 12253 | dro_anoGam | GGCCAA | 0.958 | * |  | 0 | 1 |
| CG17870 | 6610 | dro_apiMel | AACTTCTC | 0.974 |  | * | 0 | 0 |
| CG17870 | 6610 | dro_anoGam | TTAACTT | 0.923 |  | * | 0 | 0 |
| CG17888 | 13183 | dro_apiMel | AAAAAA | 0.897 |  | * | 0 | 1 |
| CG17888 | 13183 | dro_triCas | AATTAG | 1 |  | * | 0 | 0 |
| CG17888 | 13183 | dro_apiMel | ACAAAA | 0.872 |  | * | 0 | 0 |
| CG17888 | 13183 | dro_triCas | ATTAGC | 0.987 |  | * | 0 | 1 |
| CG17888 | 13183 | dro_apiMel | CAAAAA | 0.885 |  | * | 0 | 0 |
| CG17888 | 13183 | dro_triCas | CGTAAC | 0.872 | * |  | 0 | 0 |
| CG17888 | 13183 | dro_triCas | GTAACG | 0.91 | * |  | 0 | 0 |
| CG17888 | 13183 | dro2 | GTTGTT | 0.986 |  | * | 1 | 0 |
| CG17888 | 35545 | dro_triCas | AAAGTT | 0.958 |  | * | 0 | 0 |
| CG17888 | 35545 | all | CACCTA | 0.917 |  | * | 0 | 0 |
| CG17888 | 35545 | dro_apiMel | GTCGTC | 0.923 | * |  | 0 | 0 |
| CG17888 | 35545 | all | TGTCGT | 0.857 | * |  | 0 | 0 |
| CG17888 | 35545 | dro_anoGam | TGTCGTC | 0.872 | * |  | 0 | 0 |
| CG17927 | 2764 | all | CGATTA | 1 |  | * | 0 | 0 |
| CG17927 | 7684 | dro_apiMel | CTGCAA | 0.974 |  | * | 0 | 0 |
| CG17927 | 7684 | dro_apiMel | TCTGCA | 0.987 |  | * | 1 | 0 |
| CG18076 | 19937 | all | AAGTGTT | 0.833 | * |  | 0 | 0 |
| CG18076 | 19937 | dro_triCas | AGTGCCAT | 1 | * |  | 0 | 0 |
| CG18076 | 73761 | dro2 | ACTGCC | 0.972 |  | * | 0 | 0 |
| CG1830 | 5490 | dro_anoGam | GCTGAA | 0.879 |  | * | 0 | 0 |
| CG18350 | 5931 | all | ACAATAA | 0.859 | * |  | 0 | 0 |
| CG18350 | 5931 | all | CTGAGC | 0.962 |  | * | 0 | 0 |
| CG18350 | 5931 | all | GAGCGCC | 0.962 |  | * | 0 | 0 |
| CG18350 | 9304 | all | AATGTT | 1 | * |  | 0 | 1 |
| CG18350 | 9304 | all | CGAAAA | 0.983 |  | * | 0 | 0 |
| CG18408 | 19177 | dro2 | ATGTTTGC | 0.972 | * |  | 0 | 0 |
| CG18408 | 27412 | dro_apiMel | AAATGG | 0.974 | * |  | 0 | 1 |
| CG18408 | 27412 | dro_triCas | ACCCAA | 0.958 | * |  | 0 | 0 |
| CG18408 | 27412 | dro_triCas | AGAAAG | 0.821 |  | * | 0 | 0 |
| CG18408 | 27412 | dro_triCas | AGCAAGT | 0.958 | * |  | 0 | 0 |
| CG18408 | 27412 | dro_triCas | AGCCCA | 0.846 |  | * | 0 | 0 |
| CG18408 | 27412 | dro_triCas | AGTCTG | 0.859 |  | * | 0 | 0 |
| CG18408 | 27412 | dro_triCas | CGCAGC | 0.958 | * |  | 0 | 0 |
| CG18408 | 27412 | dro_anoGam | CGCTAC | 0.986 | * |  | 0 | 0 |
| CG18408 | 27412 | all | CGGTGG | 0.767 | * |  | 0 | 0 |
| CG18408 | 27412 | all | CTGGAG | 0.867 |  | * | 1 | 0 |
| CG18408 | 27412 | dro_triCas | GAGACG | 0.846 |  | * | 0 | 0 |
| CG18408 | 27412 | dro_triCas | GATCAG | 0.885 |  | * | 0 | 0 |
| CG18408 | 27412 | dro2 | GCAAGAC | 1 |  | * | 0 | 0 |
| CG18408 | 27412 | dro_triCas | GCAGCAA | 0.972 | * |  | 0 | 0 |
| CG18408 | 27412 | all | GGAGCA | 0.822 |  | * | 1 | 0 |
| CG18408 | 27412 | dro_triCas | GTCTGG | 0.885 |  | * | 0 | 1 |
| CG18408 | 27412 | all | TCTGGA | 0.833 |  | * | 1 | 0 |
| CG18408 | 27412 | all | TGGAGC | 0.844 |  | * | 0 | 0 |
| CG2049 | 12923 | all | AAGAAA | 0.885 |  | * | 1 | 1 |
| CG2049 | 12923 | dro_triCas | ATGGCA | 0.958 | * |  | 0 | 0 |
| CG2049 | 27037 | dro2 | GTTGCA | 0.958 |  | * | 0 | 0 |
| CG2049 | 27037 | dro2 | TCAGTT | 1 | * |  | 0 | 0 |
| CG2049 | 27037 | dro2 | TTCAGT | 1 | * |  | 0 | 0 |
| CG2049 | 27037 | dro2 | TTTCAG | 1 | * |  | 0 | 0 |
| CG2999 | 22844 | all | TTGAGG | 0.987 | * |  | 0 | 0 |
| CG30084 | 36532 | dro_triCas | CGCCAT | 0.936 | * |  | 0 | 0 |
| CG30084 | 36532 | dro_triCas | TCGCCA | 0.936 | * |  | 0 | 0 |
| CG30084 | 47258 | dro_triCas | GGCCTA | 0.986 | * |  | 0 | 0 |
| CG30084 | 51974 | dro2 | AACTGA | 0.986 | * |  | 0 | 0 |
| CG30084 | 51974 | dro2 | ACTGAC | 1 | * | * | 0 | 0 |
| CG30084 | 51974 | dro2 | CAACTG | 0.986 | * |  | 0 | 0 |
| CG30084 | 51974 | dro2 | GCATGC | 0.972 |  | * | 0 | 0 |
| CG30427 | 4758 | dro2 | ACGCGC | 0.958 |  | * | 0 | 0 |
| CG30427 | 4758 | all | CATCAA | 0.956 |  | * | 0 | 0 |
| CG30427 | 5251 | dro_triCas | CGGACA | 0.872 |  | * | 0 | 0 |
| CG30427 | 5251 | all | GGACAG | 0.844 |  | * | 0 | 1 |
| CG30427 | 5251 | all | GGATGC | 0.844 | * |  | 0 | 0 |
| CG30427 | 6291 | dro2 | ACCAAC | 0.972 |  | * | 1 | 0 |
| CG30427 | 6291 | dro_triCas | TCAACG | 0.897 | * |  | 0 | 0 |
| CG31000 | 17676 | dro_triCas | AAAAAT | 0.796 |  | * | 0 | 1 |
| CG31000 | 17676 | all | AAAATA | 0.767 |  | * | 0 | 1 |
| CG31000 | 59479 | all | ACGGTA | 1 | * |  | 0 | 0 |
| CG31000 | 59479 | all | AGCTGC | 0.917 |  | * | 0 | 0 |
| CG31522 | 7899 | dro_triCas | GGTTGG | 1 | * |  | 0 | 0 |
| CG32000 | 1571 | dro_triCas | CGTAATG | 0.97 | * |  | 0 | 0 |
| CG32000 | 1571 | all | GTAATG | 0.944 | * |  | 0 | 0 |
| CG32156 | 14217 | all | AGTTAC | 0.792 | * |  | 0 | 0 |
| CG32158 | 25530 | dro_triCas | AAATGA | 1 | * |  | 0 | 1 |
| CG32158 | 25530 | dro_triCas | TGCGCA | 1 |  | * | 0 | 0 |
| CG32171 | 30942 | all | GAGTCA | 0.97 | * |  | 0 | 0 |
| CG32464 | 21599 | dro2 | AGCATCA | 0.944 | * |  | 0 | 0 |
| CG32464 | 21599 | dro_anoGam | ATGAGC | 0.987 | * |  | 0 | 0 |
| CG32464 | 21599 | dro_anoGam | GAGCAT | 0.949 | * |  | 0 | 1 |
| CG32464 | 21599 | dro_anoGam | TGAGCA | 0.974 | * |  | 0 | 1 |
| CG32464 | 28127 | dro_triCas | AATATT | 0.981 |  | * | 0 | 1 |
| CG32464 | 28127 | all | AATGAA | 0.983 |  | * | 1 | 0 |
| CG32464 | 28127 | dro_triCas | AATGAAA | 1 |  | * | 0 | 0 |
| CG32464 | 28127 | all | ATGAAA | 0.983 |  | * | 0 | 1 |
| CG32464 | 28127 | all | TGAAAGAT | 0.967 |  | * | 0 | 0 |
| CG32464 | 28127 | dro_triCas | TGATAAG | 0.963 | * |  | 0 | 0 |
| CG32464 | 28127 | all | TTATCT | 0.983 | * |  | 0 | 1 |
| CG32717 | 38354 | dro2 | ATGCGC | 1 | * |  | 0 | 0 |
| CG32717 | 53880 | all | CACGCC | 0.97 | * |  | 0 | 0 |
| CG33130 | 13749 | dro_anoGam | AATGGG | 0.949 |  | * | 0 | 1 |
| CG33130 | 13749 | dro_apiMel | GAATGG | 0.974 |  | * | 0 | 0 |
| CG33130 | 13749 | dro_apiMel | GGAATG | 0.987 |  | * | 0 | 0 |
| CG33553 | 5123 | dro_anoGam | AACAAC | 0.986 |  | * | 1 | 0 |
| CG33553 | 5123 | dro_anoGam | AGCAACA | 0.972 | * |  | 0 | 0 |
| CG33553 | 5123 | dro2 | AGTTGT | 0.972 |  | * | 0 | 0 |
| CG33553 | 5123 | dro2 | ATCTCA | 0.958 |  | * | 0 | 0 |
| CG33553 | 5123 | dro_anoGam | CAACAG | 0.944 | * |  | 0 | 0 |
| CG33553 | 5123 | dro_anoGam | CAAGGA | 0.897 | * |  | 1 | 0 |
| CG33553 | 5123 | dro2 | CAGCGC | 0.944 |  | * | 0 | 0 |
| CG33553 | 5123 | dro2 | CAGTTG | 0.986 |  | * | 0 | 0 |
| CG33553 | 5123 | dro2 | GCAGTT | 0.972 |  | * | 0 | 0 |
| CG33553 | 5123 | dro2 | GGCAGT | 0.958 |  | * | 0 | 0 |
| CG33553 | 5123 | dro_anoGam | TAACAA | 0.986 | * |  | 0 | 0 |
| CG33553 | 19692 | all | AAGCAG | 0.795 |  | * | 0 | 1 |
| CG33553 | 19692 | dro_anoGam | CAGCAG | 0.872 | * |  | 0 | 0 |
| CG33553 | 19692 | all | CAGCAGC | 0.845 | * |  | 0 | 0 |
| CG33553 | 19692 | all | GCAGCA | 0.869 | * |  | 0 | 0 |
| CG33653 | 30434 | all | CCTTGA | 0.923 | * |  | 0 | 0 |
| CG33653 | 30434 | all | GAAATG | 0.905 |  | * | 0 | 1 |
| CG3399 | 15304 | dro2 | CAATGA | 0.972 |  | * | 0 | 0 |
| CG3399 | 15304 | dro2 | CCAATG | 0.972 |  | * | 0 | 0 |
| CG3399 | 15304 | dro2 | GCCAAT | 0.958 |  | * | 0 | 0 |
| CG3937 | 12352 | all | GCCGGC | 0.989 | * |  | 0 | 0 |
| CG3992 | 14988 | dro2 | ATTTAA | 1 |  | * | 0 | 1 |
| CG3992 | 14988 | dro2 | CATTTA | 1 |  | * | 0 | 0 |
| CG4300 | 2079 | all | TGGAAC | 0.9 | * |  | 0 | 0 |
| CG4898 | 5792 | dro2 | AAATGC | 0.958 | * |  | 0 | 0 |
| CG4898 | 5792 | dro_anoGam | AATCAC | 0.986 |  | * | 0 | 0 |
| CG4898 | 5792 | dro_anoGam | AGCTGTT | 0.972 |  | * | 0 | 0 |
| CG4898 | 5792 | dro_anoGam | ATCACA | 0.972 |  | * | 0 | 0 |
| CG4898 | 5792 | dro_anoGam | CTGTTT | 0.986 |  | * | 0 | 0 |
| CG4898 | 5792 | dro_anoGam | GTTTGA | 0.986 |  | * | 0 | 0 |
| CG4898 | 5792 | dro_anoGam | TGTTTG | 0.986 |  | * | 0 | 0 |
| CG4898 | 6721 | dro_apiMel | AAAAAG | 0.936 | * |  | 0 | 1 |
| CG4898 | 6721 | dro_triCas | AAAAGT | 0.944 | * |  | 0 | 1 |
| CG4898 | 6721 | dro_apiMel | AGAAAA | 0.936 | * |  | 0 | 1 |
| CG4898 | 6721 | dro_anoGam | ATGGAG | 0.949 | * |  | 0 | 0 |
| CG4898 | 6721 | all | CAAACG | 0.869 |  | * | 0 | 0 |
| CG4898 | 6721 | dro_apiMel | GAAAAA | 0.936 | * |  | 0 | 0 |
| CG4898 | 6721 | dro_triCas | GACAAC | 0.821 | * |  | 1 | 0 |
| CG4898 | 6721 | all | GCAAAC | 0.881 |  | * | 0 | 0 |
| CG5411 | 12326 | dro2 | TTGCAA | 0.986 |  | * | 0 | 1 |
| CG5670 | 13501 | all | GACGAC | 0.988 | * |  | 0 | 0 |
| CG6016 | 3998 | dro_triCas | GGGCCG | 0.833 | * |  | 0 | 0 |
| CG6016 | 3998 | dro_triCas | TGGTAT | 0.949 |  | * | 0 | 0 |
| CG6016 | 4996 | dro_apiMel | GAATTA | 0.987 | * |  | 0 | 1 |
| CG6016 | 4996 | dro_anoGam | TCGGTT | 0.987 |  | * | 0 | 0 |
| CG6043 | 491 | all | CTACGA | 0.822 |  | * | 0 | 0 |
| CG6043 | 491 | dro_apiMel | CTACGAC | 0.885 |  | * | 0 | 0 |
| CG6043 | 491 | all | TACGAC | 0.833 |  | * | 0 | 0 |
| CG6043 | 6585 | dro2 | AAGAAT | 0.958 | * |  | 0 | 1 |
| CG6043 | 6585 | dro_anoGam | AATGGC | 1 | * |  | 0 | 0 |
| CG6043 | 6585 | dro2 | AGAATC | 0.972 | * |  | 0 | 1 |
| CG6043 | 6585 | dro_anoGam | ATGGCC | 0.986 | * |  | 0 | 0 |
| CG6043 | 6585 | dro_anoGam | CAATGG | 0.958 | * |  | 0 | 0 |
| CG6043 | 6585 | dro_anoGam | TGGCCAC | 0.986 | * |  | 0 | 0 |
| CG6134 | 2573 | dro2 | TATCAA | 0.986 |  | * | 0 | 0 |
| CG6206 | 1728 | dro_triCas | CGTTTT | 0.949 |  | * | 0 | 0 |
| CG6206 | 1728 | dro_triCas | TGGATT | 0.923 | * |  | 0 | 0 |
| CG6214 | 8950 | dro_apiMel | TGCCAT | 0.987 | * |  | 0 | 0 |
| CG6214 | 9507 | dro_triCas | CTGCCA | 0.897 |  | * | 0 | 0 |
| CG6214 | 9507 | all | TGCCAC | 0.857 |  | * | 0 | 0 |
| CG7052 | 3491 | dro2 | CTGGAT | 0.985 |  | * | 0 | 0 |
| CG7052 | 4428 | all | ATATTT | 0.936 |  | * | 1 | 1 |
| CG7100 | 73981 | dro_triCas | TACCAT | 0.962 |  | * | 0 | 0 |
| CG7178 | 6218 | dro_anoGam | TTTAGA | 1 |  | * | 0 | 0 |
| CG8024 | 437 | dro2 | AAATCC | 0.958 | * |  | 0 | 0 |
| CG8024 | 437 | dro2 | CCCGCG | 0.944 |  | * | 0 | 0 |
| CG8024 | 437 | dro2 | CTGGGC | 0.944 | * |  | 0 | 1 |
| CG8024 | 437 | dro2 | GGCGCT | 0.986 |  | * | 0 | 0 |
| CG8024 | 437 | dro2 | TCTGCG | 0.986 |  | * | 0 | 0 |
| CG8201 | 8136 | dro_anoGam | CATGGG | 0.986 | * |  | 0 | 0 |
| CG8201 | 12616 | all | GTCATG | 0.944 |  | * | 1 | 0 |
| CG8295 | 1806 | dro_triCas | ACATTCC | 0.986 |  | * | 0 | 0 |
| CG8776 | 39935 | dro_anoGam | ATTGCTT | 0.972 | * |  | 0 | 0 |
| CG8776 | 39935 | dro_anoGam | GATTGC | 0.986 | * |  | 0 | 0 |
| CG8776 | 39935 | dro_anoGam | TGATTG | 0.986 | * |  | 0 | 0 |
| CG8857 | 278 | dro2 | GCGGTG | 0.944 |  | * | 0 | 0 |
| CG8857 | 278 | dro2 | GGGCTT | 0.986 |  | * | 0 | 0 |
| CG8948 | 344 | all | ATTTAT | 0.976 | * |  | 0 | 1 |
| CG9071 | 14309 | dro_triCas | GACAAG | 0.944 | * |  | 1 | 0 |
| CG9160 | 1236 | dro_anoGam | GGCAAA | 0.987 |  | * | 0 | 0 |
| CG9160 | 1236 | dro_anoGam | TGGCAA | 1 |  | * | 0 | 0 |
| CG9204 | 1346 | all | AAGGAT | 0.872 | * |  | 0 | 0 |
| CG9204 | 1346 | dro_triCas | TCCGCC | 0.936 |  | * | 0 | 0 |
| CG9614 | 36069 | dro2 | GGCAAT | 0.972 |  | * | 0 | 0 |
| CG9614 | 36069 | dro_anoGam | TCTTCA | 0.958 | * |  | 0 | 1 |
| CG9614 | 36681 | dro_triCas | ATTTCT | 0.795 |  | * | 0 | 1 |
